# Implicit intertemporal trajectories in cognitive representations of the self and nation

**DOI:** 10.3758/s13421-022-01366-3

**Published:** 2022-10-19

**Authors:** Jeremy K. Yamashiro, James H. Liu, Robert Jiqi Zhang

**Affiliations:** 1 Department of Psychology, Social Sciences 2, Room 365, University of California, Santa Cruz, 1156 High Street, Santa Cruz, CA 95064 USA; 2 Massey University, Palmerston North, New Zealand; 3 Nanjing Normal University, Nanjing, China

**Keywords:** Collective memory, Future thought, Autobiographical memory, Intertemporal trajectories, Negativity bias

## Abstract

Individual selves and the collectives to which people belong can be mentally represented as following intertemporal trajectories—progress, decline, or stasis. These studies examined the relation between intertemporal trajectories for the self and nation in American and British samples collected at the beginning and end of major COVID-19 restrictions. Implicit temporal trajectories can be inferred from asymmetries in the cognitive availability of positive and negative events across different mentally represented temporal periods (e.g., memory for the past and the imagined future). At the beginning of COVID-19 restrictions, both personal and collective temporal thought demonstrated implicit temporal trajectories of decline, in which future thought was less positive than memory. The usually reliable positivity biases in personal temporal thought may be reversable by major public events. This implicit trajectory of decline attenuated in personal temporal thought after the lifting of COVID-19 restrictions. However, collective temporal thought demonstrated a pervasive negativity bias across temporal domains at both data collection points, with the collective future more strongly negative than collective memory. Explicit beliefs concerning collective progress, decline, and hope for the national future corresponded to asymmetries in the cognitive availability of positive and negative events within collective temporal thought.

To what extent do people represent their own personal history and future as being bound with the collectivities to which they belong? And conversely, to what extent is the way people think about their group’s trajectory a function of how their own individual life is going? Both of these questions concern mentally represented intertemporal trajectories across the remembered past into the imagined future. Such trajectories may pertain to an individual’s life unfolding over time, or to a group’s historical development. Despite some overlap, cognition about one’s own past and future likely recruits different mechanisms from thinking about one’s group across time. While the procedural overlap between individual memory and imagined future events, or what we will call *personal temporal thought,* has been well established (Schacter & Addis, 2007; Schacter et al., 2012; Szpunar, 2010), research into how people think of their groups as entities extending across time, or *collective temporal thought*, is sparse in the empirical literature (Merck et al., 2016; Michaelian & Sutton, 2019; Szpunar & Szpunar, 2016). Even more scant is empirical work into how these two domains of temporal thought—personal and collective—reflect one another.

In the current studies, we infer underlying temporal trajectories implicit in people’s mental representations of themselves and their nation using retrieval fluency tasks. We examine the relation between these cognitive availability biases and explicitly endorsed beliefs and attitudes. We also examined the extent to which personal and collective trajectories track with one another. The data reported upon here were collected at two unique historical moments when personal and collective concerns came into acute alignment: the beginning of lockdowns at the start of the global COVID-19 pandemic and the subsequent return to “the new normal” 2 years later.

## Schematic temporal trajectories

### Personal trajectories

As remembered, our own lives often seem to have followed directional trajectories across time. For instance, people often believe that they have made progress relative to their past selves, even when this progress is belied by more objective measurements (Wilson & Ross, 2001). Such mental trajectories are typically structured around implicit theories concerning continuity and change (Ross, 1989). We expect some personal attributes to improve over time, others to decline, and others to follow U-shaped curves, for example. These expectations guide autobiographical recall, reconstructing memory to conform to the implicit theory. Aside from any issues of “accuracy,” such schematic trajectories provide a sense of structure and meaning to cognitive representations of the individual life across time.

### Collective trajectories

Analogous schematic trajectories also shape cognitive representations concerning the groups to which we belong. As with individual selves, people often perceive their communities to be stable entities that maintain identity and continuity across time (Sani et al., 2008a, b). This perceived continuity can serve an important psychological role in managing existential anxieties (Sani et al., 2009), as can explicit discontinuity with the collective past when that past is negative (Roth et al., 2017). As with individual self-images extending from the remembered past into the imagined future, people may represent collectives as unfolding along particular historical trajectories. For instance, people may think of their group as following a trajectory of progress, as when Martin Luther King, Jr. (1968) quoted the abolitionist Unitarian preacher Theodore Parker by claiming that the “arc of the moral universe is long, but it bends towards justice,” or, conversely, a trajectory of decline, as when the Puritan preachers and their rhetorical heirs bemoaned the moral backsliding of their colony and warned of coming retribution (Bercovitch, 1978; Murphy, 2008). At the level of mental representations, then, both individual and collective entities may follow schematic trajectories across time.

## Intertemporal thought

The cognitive and neural mechanisms involved in imagining specific episodes in the future are tightly integrated with those involved in episodic remembering (Schacter & Addis, 2007; Schacter et al., 2012; Szpunar, 2010). Research into this relation between past and future oriented *personal temporal thought* has focused almost exclusively on first-person, individual experiences. However, at least since Tulving (1985) demonstrated that the amnesic Patient K. C. could imagine events in his community’s future despite a profound inability to imagine future autobiographical episodes (see also Klein et al., 2002), the question has arisen concerning how such personal mental time travel might relate to *collective temporal thought*.

Personal temporal thought refers to an individual’s memory of their own past and imagined future. Collective temporal thought concerns collective identities and important events involving the groups with which people affiliate; it represents communities as social entities extending across time (Merck et al., 2016; Michaelian & Sutton, 2019; Szpunar & Szpunar, 2016; Yamashiro & Roediger, 2019). Collective temporal thought incorporates both collective memory (Hirst et al., 2018; Wertsch & Roediger, 2008) and imagination for the collective future (Bain et al., 2013; Szpunar & Szpunar, 2016). The integration of these two temporal domains is motivated by the argument that collective memory and collective future thought are mutually constructed in complex interactions between present goals and cultural narratives (Koselleck, 1990; Szpunar & Szpunar, 2016; Wertsch, 2002; Yamashiro & Roediger, 2019).

There are reasons, however, to believe that personal and collective temporal thought may rely on largely independent cognitive processes. Collective temporal thought, for instance, typically does not involve autonoetic consciousness, one of the defining features of episodic temporal thought (Manier & Hirst, 2008). Autonoetic consciousness is the self-aware feeling of reexperiencing (or preexperiencing) an event from the first person—or “field”—perspective, and associated *mental time travel* (Tulving, 1972). Temporal thought in general, however, may incorporate episodic and semantic elements to different degrees (Szpunar, 2010); at one limit, for instance, an imagined future event may involve no episodic content at all.

Collective temporal thought may thus lack the prototypical episodic features of personal temporal thought while, nevertheless, sharing some overlapping features, such as implicit theory driven schematic construction. Collectively represented intertemporal trajectories are of interest in that they may shape the conditions for collective identification and collective action (Shteynberg et al., 2020). For instance, imagining a benevolent collective future has been associated with personal actions in the present to produce such a future (Bain et al., 2013).

## Implicit intertemporal trajectories

### Specific narratives and schematic temporal trajectories

 We have proposed that the relation between memory and imagination for the future is often mediated by theories about temporal change, which can take the form of schematic trajectories across time. Before discussing how one might study such trajectories empirically, we must clarify our definition of an *implicit temporal trajectory*. We distinguish explicit *specific narratives* and implicit *schematic temporal trajectories*. This approach draws from Wertsch’s (2002) distinction between specific narratives and schematic narrative templates. Specific narratives of, say, progress, might include the passage of the Voting Rights Act of 1965 and its role in advancing political equality for African Americans, or of a particular junior scholar in a prestigious academic program overcoming her imposter syndrome to develop self-confidence in her scholarly aptitude. These specific narratives have concrete settings, agents and antagonists, and other necessary plot elements; they are potentially available to conscious awareness if one knows the story, and they can be communicated to others.

On the other hand, as we use the term, implicit temporal trajectories are schematic representations of progress or decline, generically. They correspond to a subset of Ross’s (1989) implicit theories of change, theories in which things, in general, are better now than they used to be (or vice versa). While the schematic trajectory of progress might be said to underlie both the stories about the Voting Rights Act and the junior scholar’s increasing confidence, implicit temporal trajectories do not themselves possess any of the concrete details required for specific narratives—the who, where, when, why, how (Wertsch, 2021). As schemata, such implicit trajectories may be used to *emplot* a variety of different specific narratives (Ricoeur, 1991). They provide a culturally shared scaffolding to structure and give socially recognizable meaning to particular narratives. Referring to one characteristically Russian schematic narrative template, for instance, Wertsch (2002) demonstrates how the abstract “expulsion of alien enemies” plot is deployed in telling many specific events in Russian collective memory and contemporary understanding of geopolitics.

Schematic narrative templates are not directly observable in the way specific narratives are. They can, however, be inferred from common patterns in the way people tell personal or collective stories or, as we shall show in the current studies, in the way such schemata scaffold recall for schema consistent events and decrease accessibility for schema inconsistent events (Anderson & Pichert, 1978; Hirst & Yamashiro, 2017). Importantly, these cultural tools for thought are cognitively transparent; people tend not to be consciously aware of using them (Wertsch, 2021). As such, they are typically implicit phenomena. However, we can use cognitive methods for tracing their outlines and measuring their influence. If it is the case that people employ implicit temporal trajectories of progress or decline while thinking about the past and future, representations that are congruent with the proposed schematic trajectory should, all else being equal, be more cognitively accessible, subjectively meaningful, and intuitive feeling than representations that are incongruent with the schema (Yamashiro, 2021).

### Detecting implicit temporal trajectories

Evidence for an implicit temporal trajectory can be indicated by a change in the degree or direction of bias in the emotional valence of cognitive representations pertaining to at least two separate temporal periods. These two periods are typically the past and the future, although some researchers have fruitfully examined implicit temporal trajectories across multiple periods in the past, for example, in Belgian representations of colonialism and decolonization of the Congo (e.g., Lastrego et al., 2022). The relative fluency with which people retrieve events from a target period provides a behavioral measure of cognitive accessibility (i.e., the speed or ease with which representations from a particular domain come to mind). Valenced biases occur when negative and positive events from a target period are differentially accessible to recall. Retrieval fluency-based tests for valenced biases were initially developed to detect clinically relevant biases in autobiographical memory and personal future thought (e.g., MacLeod & Byrne, 1996; MacLeod et al., 1993, 1997). The general approach has since been adapted to examine affective biases in collective memory and collective future thought.

The first such studies focused exclusively on collective future thought. Shrikanth et al.’s (2018) participants engaged in a timed retrieval fluency task in which they listed events for which they were either excited or worried about in their personal future or their country’s future. Participants showed a domain (personal, collective) by valence (negative, positive) interaction, in that they tended to show positivity biases when thinking about their personal future and pronounced negativity biases when thinking about events in their country’s future. This interaction replicated in both American and Canadian samples, in near and distant future thought, and across political affiliations in the American sample. From this evidence, Shrikanth et al. (2018) concluded that personal and collective future thought were dissociable domains of future thought—that is, that collective future thought is not reducible to the impact people believe collective events will have on themselves personally. A similar dissociation has been found between collective and autobiographical memory (Shrikanth & Szpunar, 2021).

Subsequent research stressed the need to interpret such valenced biases in the context of broader intertemporal trajectories. Yamashiro and Roediger (2019) extended Shrikanth et al.’s (2018) retrieval fluency paradigm to include, in addition to collective future thought, collective memories for national origins, and normative collective memories (i.e., the national events Americans believe their fellow Americans ought to remember). They replicated Shrikanth et al.’s (2018) negativity bias in collective future thought. However, rather than a symmetrical negativity bias in collective memory, the “dystopian” collective future was imagined in counterpoint to pronounced positivity biases in at least two domains of collective memory. The juxtaposition of positivity biases in collective memory and negativity biases in collective future thought suggested an implicit trajectory of decline in Americans’ representations of their nation. This trajectory of decline relative to national origins has been replicated in a French sample (Ionescu et al., 2022). As a caveat, national origins and normative collective memory may be special domains for which there are cultural and social pressures to produce positive representations (Topçu & Hirst, 2022; Yamashiro et al., 2019; for cross-cultural evidence of positivity biases in national origin stories, see Choi et al., 2021). Outside of these special domains, negativity biases in collective memory may be more common (Hacibektasoglu et al., 2022; Öner & Gülgöz, 2020; Topçu & Hirst, 2020), especially as they frequently contain collective memories of war (Liu & Páez, 2019).

## The current study

We collected two samples, from the United States of America (USA) and the United Kingdom (UK). The choice of these two countries was not meant as a cross-cultural comparison so much as a test of generality. Although several studies have now demonstrated the relative cognitive independence of personal and collective temporal thought, the extent to which personal and collective implicit temporal trajectories reflect one another remains poorly understood. We thus examined whether there is a relation between implicit temporal trajectories in personal and collective temporal thought.

Additionally, we examined the relation between valenced asymmetries in retrieval fluency and explicit attitudes about collective progress and decline. While the cognitive accessibility of an event does indeed inform people’s estimate of how likely it is to happen (Tversky & Kahneman, 1973), and people do use asymmetries in the cognitive availability of historical events in memory to inform judgments about their groups’ historical influence (Yamashiro & Roediger, 2020), the relation between implicit temporal trajectories and explicit beliefs has yet to be specified.

## Methods

### Participants

Participants in the USA and UK were recruited through Toluna, an international market research company, as part of a larger survey reported elsewhere (Zhang et al., 2022). The samples were stratified on age, gender, and region. Data were collected between March 26 and April 8, 2020. In total, we obtained 1,155 survey responses. From this initial sample, 243 participants were screened for providing nonsense answers in the retrieval fluency tasks (USA = 147, UK = 96), leaving a final sample of 912 participants. Of the American sample (*N* = 435), 63% of the sample identified as female, 34% as male, and 2% as other. Of the British sample (*N* = 477), 58% identified as female, 40% as male, and 1% as other. The two samples did not differ in mean age, *p* = .27, which was 51.1 years old, with a range from 18 to 88. A G*Power analysis indicated that for the most demanding planned test, 82 participants were required for proper power; our sample adequately surpassed this. The G*Power protocol is provided in the Open Science Framework (OSF) repository linked in the Declarations.

### Procedures

Participants engaged in a series of eight timed retrieval fluency tasks, programmed into a single Qualtrics survey. The cue for each retrieval task targeted one of eight cells: 2 (referent: self, nation) × 2 (temporal period: past, future) × 2 (valence: positive, negative). For example, participants might see the cue “Negative events in your own personal past,” to which they were to list as many negative autobiographical memory events as they could within 1 minute, each separated by a comma. Cues for each of the eight target cells appeared individually, one per page. The order of the eight cues was randomized for each participant, and all participants received all eight cues. The survey automatically advanced participants after they had spent one minute typing their response to each cue; participants could not self-advance. Participants in the USA and UK followed identical procedures, except that the name of their country in the National Referent cues was matched appropriately.

### Measures

#### Retrieval fluency measures

The full set of protocols was dual coded by independent raters; interrater reliability was nearly perfect (Cronbach’s α = .99), and all subsequent analyses were conducted using the codes from the first rater. Of primary interest was the positivity/negativity bias for different referents (self, nation) in different temporal periods (past, future). A positivity bias would be evident if positive events were more accessible (i.e., more had been listed relative to negative events), and vice versa for a negativity bias. In the first set of analyses, the simple count of discrete events provided in each of the eight cells was the dependent variable of interest.

#### Proportion positive

In the second set of analyses, in order to examine implicit intertemporal trajectories in representations of the self and nation, we calculated *proportion positive* for a particular referent and temporal period, which is the number of positive events over the sum of negative and positive events. For example, for autobiographical memory (ABM), Proportion Positive_*ABM*_ = Count_*ABM:Positive*_ / **∑** (Count_*ABM:Positive*_*,* Count_*ABM:Negative*_), and analogously for personal future thought (PFT), collective memory (CM), and collective future thought (CFT). This measure standardizes the bias measure across different levels of output productivity. We define a valenced bias as a proportion positive that deviates significantly from the criterion value of .5, which would indicate that positive and negative events were equally accessible.

#### Explicit belief measures

Participants indicated agreement on a 6-point scale, ranging from from *Strongly Agree* to *Strongly Disagree*, for 10 explicit beliefs concerning national trajectories: *I am* [*Hopeful/Worried*] *about my nation’s future*; *I am* [*Proud/Ashamed*] *of my nation’s history*; *Relative to my nation at its founding, things are* [*Better/Worse/About the Same*] *now*; *My nation is experiencing* [*Progress/Decline*]; and *My nation is now about the same as it has always been*. Explicit belief ratings always came after the retrieval fluency tasks.

## Results

All number pairs in brackets represent 95% confidence intervals. Post hoc tests with multiple comparisons are presented with Tukey adjustments unless otherwise indicated. For clarity of exposition, we first present the findings for personal temporal thought and collective temporal thought separately, finishing with the relation between the two.

### Personal temporal thought

Americans and Britons did not differ in any domain of personal temporal thought, with the main effect and interactions involving country all *p*s > .07. The two national samples are thus collapsed in Table 1, which presents referent-by-valence item counts for personal temporal thought. To compare retrieval fluency for negative and positive events, we conducted a 2 (temporal period: past, future) × 2 (valence: positive, negative) × 2 (country: USA, UK) mixed analysis of variance (ANOVA) on the number of discrete events participants produced during the timed retrieval fluency task.

**Table 1:** Study 1: Event counts for timed retrieval fluency task on personal temporal thought

Valence	Temporal Period	
	Autobiographical Memory	Personal Future Thought
Positive	4.05 [3.93, 4.17]	3.08 [2.96, 3.21]
Negative	3.19 [3.07, 3.32]	3.21 [3.09, 3.33]

Bracketed numbers represent 95% CIs

Autobiographical memories were produced more fluently than personal future thoughts in a main effect for temporal period, *F*(1, 910) = 110.73, *p* < .001, and positive events were produced more fluently than negative events in a main effect for valence, *F*(1, 910) = 57.22, *p* < .001. However, these main effects were qualified by an interaction between temporal period and valence, *F*(1, 910) = 140.78, *p* < .001. Replicating some prior reports (Yamashiro & Roediger, 2019) but at odds with others (Shrikanth et al., 2018), autobiographical memory showed a positivity bias, *M*_diff_ = 0.86, *SE* = 0.06, *t*(1779) = 13.47, *p* < .001, *d* = 0.45, while personal future thought showed no bias in emotional valence, *M*_diff_ = 0.13, *SE* = 0.06, *t*(1779) = 1.98, *p* = .20, *d* = 0.07. This pattern emerged because although negative events were equally accessible between autobiographical memory and personal future thought, *p* = .99, positive events were relatively less accessible in personal future thought than in autobiographical memory, *M*_diff_ = .97, *SE* = .06, *t*(1807) = 15.78, *p* < .001, *d* = 0.54. Age correlated negatively with retrieval fluency; as age increased, participants produced both fewer positive future events, *r*(894) = −.20 [−.26, −.14], *p* < .001, and fewer negative events *r*(894) = −.07 [−.14, −.01], *p* = .03.

#### Proportion positive

Data for the analyses on proportion positive in personal temporal thought are presented in Table 2.

**Table 2:** Study 1: Personal temporal thought—Proportion positive across temporal periods, by country

Country	Temporal Period	
	Autobiographical Memory	Personal Future Thought
USA	.55 [.53, .57]	.48 [.47, .50]
UK	.57 [.55, .59]	.47 [.45, .49]

The 95% Confidence intervals (bracketed numbers) that do not straddle .50 indicate a valenced bias

We tested first for valenced biases using one-sampled *t* tests against a criterion value of .5. There was a positivity bias in autobiographical memory (proportion positive *M* = .56 [.55, .57]), *M*_diff_ from .5 = .06 [.05, .07]), *t*(907) = 10.72, *p* < .001, *d* = 0.36. There was also a small but statistically significant negativity bias in personal future thought (*M* = .48 [.47, .49]), *M*_diff_ from .5 = −.02 [−.04, −.01]), *t*(902) = 3.42, *p* < .001, *d* = 0.11. To test for implicit temporal trajectories of personal progress or decline, we submitted proportion positive to a 2 (temporal period: autobiographical memory, personal future thought) × 2 (country: USA, UK) mixed ANOVA. There was a main effect of temporal period suggesting an implicit trajectory of decline, *F*(1, 897) = 95.63, *p* < .001, with autobiographical memory (*M* = .56 [.55, .57]) proportionally more positive than personal future thought (*M* = .48 [.47, .49]), *M*_diff_ = −.08 [−.10, −.07], *t*(898) = 9.87, *p* < .001, *d* = 0.32. There was no main effect of country on proportion positive, *p* =.55, but country did interact with temporal period, *F*(1, 897) = 4.57, *p* = .03. Personal implicit temporal trajectories had different gradients between the American and British samples. The decline in positivity from autobiographical memory to personal future thought was steeper in the UK sample, *M*_diff_ = .10, *SE* = .01, *t*(891) = 8.64, *p* < .001, *d* = 0.46, than in the USA sample, *M*_diff_ = .06, *SE* = .01, *p* < .001, *d* = 0.29, although both showed trajectories of decline. This was accounted for in the noncorrected post hoc by the fact that British autobiographical memory (*M* = .57, *SE* = .01) was slightly more positive than American autobiographical memory (*M* = .55, *SE* = .01), *M*_diff_ = .02 [0, .05], *t*(906) = 2.02, *p* = .04, *d* = 0.13, but this post hoc test did not survive the application of the Tukey correction for multiple comparisons, *p* = .24. The two countries did not differ in the proportion positive of personal future thought, *p* = .76.

#### Proportion positive and age

The degree of positivity decline from autobiographical memory to personal future thought correlated with age, *r*(883) = .14 [.07, .20], *p* < .001. This dynamic was largely driven by the fact that the positivity of personal future thought decreased with age, *r*(887) = −.11 [−.18, −.04], *p* < .001, along with a nonstatistically significant effect of autobiographical memory becoming slightly more positive with age, *r*(890) = .06 [−.002, .13], *p* = .06. Proportion positive in autobiographical memory and personal future thought correlated with one another positively, but rather weakly, *r*(897) = .11 [.04, .17], *p* = .002, suggesting some individual difference tendencies toward overall positivity or negativity.

### Collective temporal thought

We next examined intertemporal representations of the nation. As with personal temporal thought, we first present data on raw event counts from the fluency tasks (see Table 3), then on the proportion positive measure across temporal periods (see Table 4). The USA and UK samples did differ in collective temporal thought, and so are not collapsed in these analyses.

**Table 3:** Study 1: Event counts for timed retrieval fluency task on collective temporal thought

Country	Valence	Temporal Period	
		Collective Memory	Collective Future Thought
USA	Positive	2.58 [2.42, 2.74]	2.04 [1.88, 2.20]
	Negative	3.69 [3.53, 3.85]	2.99 [2.83, 3.15]
UK	Positive	2.47 [2.31, 2.63]	1.64 [1.49, 1.80]
	Negative	3.02 [2.86, 3.17]	2.86 [2.70, 3.02]

Bracketed numbers represent 95% CIs

**Table 4:** Study 1: Collective temporal thought—Proportion positive across temporal periods, by country

Country	Temporal Period	
	Collective Memory	Collective Future Thought
USA	.41 [.39, .43]	.39 [.37, .41]
UK	.45 [.43, .47]	.34 [.32, .36]

Participants represented their national past in greater detail than their national future, more fluently providing collective memories (*M* = 2.94 [2.85, 3.03]) than collective future thought events (*M* = 2.38 [2.29, 2.48]), *M*_diff_ = 0.57, *SE* = .04, *t*(897) = 13.2, *p* < .001, *d* = 0.47, as shown in a main effect for temporal period, *F*(1, 897) = 173.03, *p* < .001. However, whereas personal temporal thought had shown an overall positivity bias, collective temporal thought showed a negativity bias, with the main effect of valence, *F*(1, 897) = 433.68, *p* < .001, indicating that participants could more fluently think of negative (*M* = 3.14 [3.04, 3.24]) than positive (*M* = 2.18 [2.09, 2.28]) events, *M*_diff_ = .0.96, *SE* = .05, *t*(897) = 20.8, *p* < .001, *d* = 0.64. There was a main effect of country, with Americans (*M* = 2.83 [2.71, 2.95]) providing more events than British (*M* = 2.50 [2.38, 2.62]), *M*_diff_ = 0.33, *SE* = .09, *t*(897) = 3.81, *p* < .001, *d* = 0.12.

While both collective memories and collective future thought demonstrated negativity biases, the two temporal periods differed in the strength of that bias, as indicated in a temporal period by valence interaction, *F*(1, 897) = 10.36, *p* = .001. The negativity bias was stronger in collective future thought, *M*_diff_ = 1.08, *SE* = 0.06, *t*(1756) = 17.88, *p* < .001, *d* = 0.60, than in collective memory, *M*_diff_ = 0.83, *SE* = .06, *t*(1756) = 13.68, *p* < .001, *d* = 0.46, again suggesting an intertemporal trajectory of decline. This temporal period by valence interaction differed by country in a three-way interaction, *F*(1, 897) = 27.25, *p* < .001. To unpack this three-way interaction more clearly, we examined the proportion positive analyses.

#### Proportion positive

There was a negativity bias in both collective memory (*M* = .43, *SE* = .007), *M*_diff_ from .50 = −.07 [−.08, −.05], *t*(877) = 9.89, *p* < .001, *d* = 0.33, and an even stronger negativity bias in collective future thought (*M* = .36, *SE* = .008), *M*_diff_ from .50 = −.14 [−.15, −.12], *t*(880) = 17.47, *p* < .001, *d* = 0.59. Comparing proportion positive across temporal periods, there was an implicit trajectory of decline, *F*(1, 859) = 74.6, *p* < .001, with collective future thought (*M* = .36, *SE* = .008) less positive than collective memory (*M* = .43, *SE* = .007), *M*_diff_ = .07 [.05, .09], *t*(857) = 7.01, *p* < .001, *d* = 0.24. While there was no main effect of country, *p* = .63, there was an interaction between country and temporal referent, *F*(1, 856) = 17.9, *p* < .001. For the USA, proportion positive did not change statistically from collective memory to collective future thought, *p* = .25, although the numerical difference was in the direction of a slight decline. The UK showed a decline trajectory, *M*_diff_ = .11, *SE* = .02, *t*(856) = 8.04, *p* < .001, *d* = 0.18. This was accounted for by the fact that the UK had numerically, though not statistically, more positive collective memory than the USA, *M*_diff_ = .04, *SE* = .01, *t*(1693) = 2.47, *p* = .07, *d* = 0.13, and less positive collective future thought than the USA, *M*_diff_ = .05, *SE* = .01, *t*(1693) = 3.18, *p* = .008, *d* = 0.17. Finally, implicit trajectories in personal temporal thought correlated, albeit weakly, with implicit trajectories in collective temporal thought, *r*(845) = .08 [.008, .14], *p* = .028.

### Explicit beliefs measures

Valenced biases in retrieval fluency did tend to correlate as expected with explicitly endorsed beliefs (see Table 5). This was in line with prior work that the cognitive availability of events in collective memory informs evaluative judgments about the collective (Yamashiro & Roediger, 2020), extending this finding to collective future thought and, to a more limited extent, to implicit temporal trajectories. While valenced biases in both temporal domains correlated with explicit attitudes, valenced biases in collective future thought tended to correlate more strongly with future oriented beliefs (i.e., hope or worry about the future of the nation, belief in progress or decline). Although there was some indication of an analogous relation between collective memory and past oriented attitudes (e.g., biases in collective memory correlated somewhat more strongly with pride in the nation’s history than did biases in collective future thought), this effect in past oriented collective temporal thought was less apparent. Relations between valenced biases in personal temporal thought and explicitly endorsed attitudes about the nation partially mirrored those between collective temporal thought and explicit attitudes, though more weakly. Of interest, although most correlations between implicit temporal trajectories (i.e., the change in valenced bias between memory and future thought) and explicitly endorsed beliefs were either very weak or nonsignificant, one belief did correlate moderately with the change in valence bias: Hopefulness about the future of the nation.

**Table 5:** Study 1: Correlations between explicit attitudes and valenced retrieval biases

	Valence Bias				Change in Valence Bias	
	ABM	PF	CM	CF	PTT	CTT
Hopeful	.10**	.16***	.17***	.33***	.06	.15***
Worried	−.10**	−.16***	−.16***	−.20***	−.07*	−.05
Proud	.06	.05	.18***	.15***	−.01	.003
Ashamed	−.07*	.01	−.09**	−.11**	.05	−.03
Better	.02	.09**	.11*	.09**	.06	−.006
Worse	−.007	−.09**	−.10**	−0.06	−.07*	.01
Progress	.084*	.10**	.15***	.20***	.03	.05
Decline	−.094**	−.06	−.15***	−.22***	.01	−.07*
Same	.02	.06	.05	.09**	.04	.03
Stasis	.00	.03	.04	.01	.02	−.03

*ABM* Autobiographical Memory, *PF* Personal Future, *CM* Collective Memory, *CF* Collective Future, *PTT* Personal Temporal Thought, *CTT* Collective Temporal Thought

^*^ *p* < .05, ** *p* < .01, *** *p* < .001

## Study 1 discussion

Regarding the question of the extent to which personal and collective trajectories track with one another, the answer seems to be that although there is some relation, the relation is quite weak. The weakness of this correlation between personal and collective implicit temporal trajectories is in line with prior work in Western countries indicating that personal and collective temporal thought are largely dissociable domains of thought (Shrikanth & Szpunar, 2021; Shrikanth et al., 2018; Yamashiro & Roediger, 2019). However, the collective temporal thought negativity bias replicated robustly, both in terms of the collective future thought negativity bias and in the implicit temporal trajectory of national decline. This is the first demonstration of these phenomena in a British sample, adding the UK to the list of Western democracies in which an implicit trajectory of decline has been demonstrated in mental representations of the nation.

Aligning with reports that representations of the personal past are more phenomenologically vivid than representations of the personal future (D’Argembeau & Van der Linden, 2004, 2006), our fluency measures indicated that memories were on average more accessible than imagined future events, for both the personal and collective referents. We likewise replicated the frequently reported finding of a positivity bias in autobiographical memory. However, this positivity bias did not extend across all domains of personal temporal thought. In contrast to prior research showing positivity biases in personal future thought (Newby-Clark & Ross, 2003; Shrikanth et al., 2018), our participants showed either no valenced bias in personal future thought (using the fluency count measure) or a negativity bias (using the proportion positive score).

Such a personal future thought negativity bias is unusual in the literature. One possible explanation could be that, given the reliable negativity bias in collective temporal thought, thinking of the personal future in the context of collective events primed negativity. Another likely contributor was the historical moment in which these data were collected. Study 1 began right as COVID-19 lockdowns took effect in both the USA and UK; it would not be unreasonable to suppose that this profound disruption to normal life would overcome the normally robust personal future thought positivity bias. Indeed, a perfunctory content analysis of the retrieval fluency protocols suggests that COVID-19 and the measures taken to control its propagation loomed large in our participants’ representations of the future—at least 950 direct mentions of the coronavirus or pandemic in a sample of 912, and many other allusions to the pandemic in the form of worries about the economic impacts of lockdowns, social distancing, access to health care, and other disruptions to ordinary life. Study 2 was designed to examine the contribution of these two possible explanations for the personal future thought negativity bias. Study 2 also allowed us to examine how stable the collective temporal thought negativity bias and implicit trajectories of decline were across time.

## Study 2

Whereas the initial sample was collected at the onset of lockdowns for the COVID-19 pandemic, the Study 2 sample was collected at what was widely perceived as the beginning of the “return to the new normal,” in April 2022. At this point, vaccines and boosters had become widely available and many businesses and workplaces were beginning to resume relatively normal business in both the USA and UK. Although globally the pandemic continued, we surmised that Britons and Americans, at least, may have begun to imagine a more optimistic future than was the case at the beginning of the pandemic. If this were the case, we would expect to see the typical personal future thought positivity bias return.

Study 2 further allowed us to test more directly whether thinking of personal events in the context of collective events impacted valenced biases in personal temporal thought. In Study 1, personal temporal thought cues had been interleaved with collective temporal thought cues. Given a reliable collective temporal thought negativity bias, it was possible that such interleaving primed negativity on the personal temporal thought tasks. That is, the personal future may have become more negative when people thought about it in the context of collective events. Thus, we additionally tested for whether interleaving versus segregating personal and collective temporal thought probes impacted the observed biases.

## Methods

### Participants

A G*Power analysis indicated that detecting a valence-by-temporal referent-by-task order interaction required a total sample size of 98. We collected 101 participants via a Prolific standard sample, selecting at random from the USA and UK. Because of the time of day at which the survey was launched, the UK sample predominated (UK *N* = 78, USA *N* = 23). Thus, in this sample, between-country comparisons should be treated with caution. However, the country factor was not central to our purposes for Study 2, which were primarily focused on the impact of historical moment and the possibility of task order effects in explaining our previous findings. Participants had a mean age of 34 years old, with a range from 18 to 89. Gender breakdown of the sample was 62% female, 34% male, and 4% other. The sample was relatively well educated, with 29.7% with some college, 31.7% with a 4-year degree, 13.9% with a professional degree, 17.8% with a high school degree, 5.9% with a 2-year degree, and 1% with less than high school. Ethnically the sample was 76.2% White, 13.9% Asian, 5% Black, 3% Other, and all other identifiers below 1%.

### Procedures

The retrieval fluency and explicit ratings tasks were identical to Study 1, with the following exceptions. Retrieval fluency/explicit rating task order was randomized across participants. Additionally, within the fluency tasks, participants could receive one of two conditions: interleaved or ordered. In the interleaved condition, which was a direct replication of Study 1 procedures, the eight fluency probes—that is, 2 (referent: personal, collective) × 2 (temporality: past, future) × 2 (valence: positive, negative)—were fully randomized for each participant. In the ordered condition, participants saw all four personal referent probes together, then all four collective referent probes together, or vice versa, with referent (i.e., personal or collective) order randomized for each participant. The interleaved probe condition and the collective temporal thought probes first ordered condition provided two potential ways for collective temporal thought to prime negativity in personal temporal thought. These two forms of collective temporal thought priming could be compared with the personal temporal thought first ordered condition, in which personal temporal thought probes were presented prior to any “contamination” by collective temporal thought. Of the *N* = 101 full sample, 50 participants received interleaved probes, and 51 received ordered probes. Of the participants receiving ordered probes, 27 received collective then personal temporal thought probes, and 24 received personal then collective temporal thought probes. Finally, Study 2 data were collected in April 2022, at a period when most pandemic restrictions had been lifted in the USA and UK, which allowed us to test the extent to which the biases observed in Study 1 were the result of the unusual historical moment in which the original data were collected.

## Results

### Retrieval fluency tasks

As in Study 1, we examine personal temporal thought first, then collective temporal thought, then the relation between them.

#### Personal temporal thought

For personal temporal thought data, see Table 6. Although numerically the Study 1 patterns were largely repeated, retrieval fluency was not statistically impacted by temporal period, *p* = .32, or valence, *p* = .74, and there was no interaction between temporality and valence, *p* = .09. The hypothesis that the personal future thought negativity bias found in Study 1 was due to interleaving personal with collective temporal thought probes was not supported; interleaving versus segregating personal and collective temporal thought probes produced no main effect, *p* = .069, nor any interactions, all *p*s > .05. Similarly, in the segregated probe conditions, responding to collective vs. personal temporal thought probes first produced no main effect, *p* = .35, nor any interactions, all *p*s > .05.

**Table 6:** Study 2: Event counts for timed retrieval fluency task on personal temporal thought

Valence	Temporal Period	
	Autobiographical Memory	Personal Future Thought
Positive	4.55 [4.15, 4.98]	4.16 [3.67, 4.66]
Negative	4.37 [3.93, 4.81]	4.47 [4.02, 4.92]

Bracketed numbers represent 95% CIs.

#### Collective temporal thought

The findings for collective temporal thought largely replicated the findings from Study 1. On average participants could think of past events (*M* = 3.64 [3.32, 3.95]) more fluently than future events (*M* = 3.21 [2.90, 3.53]), *M*_diff_ = .43, *SE* = .13, *F*(1, 100) = 11.65, *p* < .001. There was also a general negativity bias, with negative events (*M* = 4.16 [3.84, 4.49]) more accessible than positive events (*M* = 2.69 [2.36, 3.02]), *M*_diff_ = 1.48, *SE* = .16, *F*(1, 100) = 88.39, *p* < .001. As before, there was also a temporality by valence interaction, *F*(1, 100) = 6.58, *p* = .01. Whereas there were negativity biases in both collective memory and collective future thought, the negativity bias in collective future thought, *M*_diff_ = 1.89, *SE* = .22, *t*(200) = 8.47, *p* < .001, was greater than the negativity bias in collective memory, *M*_diff_ = 1.06, *SE* = .22, *t*(200) = 4.74, *p* < .001. This new sample thus replicates the implicit trajectory of decline in mental representations of the nation.

#### Task order interactions

While task order effects did not explain the unusual negativity bias in personal future thought, they did impact collective temporal thought. There was a main effect of task order, *F*(1, 99) = 6.61, *p* = .012, with collective temporal thought events retrieved more fluently after explicit evaluative reflection on the nation’s trajectory than before such reflection, *M*_diff_ = 0.71, *SE* = 0.28, *t*(99) = 2.57, *p =* .012. This effect of task order interacted with temporality, *F*(1, 99) = 9.03, *p* < .001. Reflecting explicitly on the trajectory of one’s nation seemed to increase the relative accessibility of collective memories, but not collective future thoughts. Collective memories were produced more fluently after explicitly reflecting on the nation’s trajectory (*M* = 4.19, *SE* = .22) than when the retrieval fluency task happened before such explicit reflection (*M* = 3.01, *SE* = .22), *M*_diff_ = 1.09, *SE* = 0.31, *t*(133.2) = 3.54, *p =* .003. Interestingly, this priming did not occur for collective future thought, where task order did not impact fluency, *M*_diff_ = 0.37, *SE* = 0.31, *t*(133.2) = 1.20, *p =* .63.

Valenced biases were not impacted by task order, as indicated by no Temporality × Valence × Task Order interaction, *p* = .41. There were no main effects of interleaving versus segregating personal and collective temporal thought probes on fluency of retrieval, *p* = .46, nor any interactions, all *p*s > .36. Similarly, in the ordered condition, collective versus personal probes first produced no main effect, *p* = .75, nor any interactions, all *p*s > .08. Thus, interleaving vs. segregating personal and collective temporal thought probes had no impact on the valence biases for either personal or collective referents, in either temporal domain.

### Proportion positive

We next examined proportion positive, calculated in the same way as in Study 1.

#### Personal temporal thought

In contrast to Study 1, neither autobiographical memory (*M* = .52, *SE* = .01), nor personal future thought (*M* = .47, *SE* = .02), demonstrated a valenced bias, operationalized as a significant difference from a criterion value of .5, *M*_diff_ = .02 [−.002, .05], *t*(100) = 1.79, *p =* .077, and *M*_diff_ = −.03 [−.07, .002], *t*(100) = 1.86, *p =* .066, respectively. However, an implicit trajectory of decline did replicate, with autobiographical memory more positive than personal future thought, *M*_diff_ = 0.05 [.02, .09], *t*(100) = 2.78, *p =* .006, *d* = 0.28. Overall, 58% of participants showed a decline trajectory in personal temporal thought, operationalized as an autobiographical memory that was more positive than personal future thought. There were no correlations with age, all *p*s > .05, and no main effects or interactions with country, all *p*s > .05. Task order produced no main effects or interactions, all *p*s > .05, again suggesting valence biases in personal temporal thought were not impacted by engaging in personal temporal thought in the context of collective temporal thought.

#### Collective temporal thought

Replicating Study 1, both collective memory (*M* = .43, *SE* = .018) and collective future thought (*M* = .32, *SE* = .02) demonstrated negativity biases, differing significantly from the criterion value of .5, Collective Memory *M*_diff_ = −0.07 [−.11, −.04], *t*(100) = 4.23, *p <* .001; Collective Future Thought *M*_diff_ = −.18 [−.22, −.14], *t*(100) = 8.57, *p <* .001. As before, there was an implicit trajectory of decline in collective temporal thought, with the collective future more negative than collective memory, *M*_diff_ = .11 [.05, .16], *t*(100) = 3.73, *p =* 0.37. Overall, 65% of participants showed a decline trajectory in collective temporal thought. There were no correlations with age, all *p*s > .05, and country did not interact with temporality, *p* = .25. As with the direct fluency measures, task order produced no main effects or interactions, all *p*s > .05.

Finally, the implicit temporal trajectories for personal and collective temporal thought did not correlate significantly, *r*(99) = .16 [−.04, .35], *p* = .11.

### Explicit belief measures

Table 7 provides a correlation matrix between explicit beliefs and the proportion positive measures.

**Table 7:** Study 2: Correlations between explicit measures and proportion positive and implicit trajectories of change

	Valence Bias				Change in Valence Bias	
	ABM	PF	CM	CF	PTT	CTT
Hopeful	.12	.43***	.06	.47***	.31**	.32**
Worried	−.08	−.19	−.05	−.14	−12	−.07
Proud	−.02	.18	.07	.22*	.17	.12
Ashamed	.03	−.13	−.03	−.25*	−.13	−.17
Better	−.04	.15	.17	.14	.16	−.004
Worse	.006	−.25*	−.19	−0.16	−.23*	−.003
Same	.09	.12	.11	.005	.04	−.07
Progress	−.008	.23*	.08	.34***	.21*	.20*
Decline	−.02	−.38***	−.12	−.32**	.33***	−.16
Stasis	.09	.11	−.1	.09	.04	.13

*ABM* Autobiographical Memory, *PF* Personal Future, *CM* Collective Memory, *CF* Collective Future, *PTT* Personal Temporal Thought, *CTT* Collective Temporal Thought

^*^ *p* < .05, ** *p* < .01, *** *p* < .001

Valenced biases in retrieval correlated with explicitly expressed beliefs largely along the lines found in Study 1. Proportion positive in both the personal and collective future correlated most strongly with hope towards the nation’s future, and also moderately with belief in national progress. Proportion positive in both personal and collective future thought correlated negatively with belief in national decline. Again, the implicit trajectory correlated with explicitly endorsed hopefulness regarding the nation’s future, as well as with explicit belief in progress. Interestingly, belief in national decline also correlated robustly with the personal temporal trajectory.

## Study 2 discussion

Study 2 had two primary aims: to examine whether the unusual personal future thought negativity bias found in Study 1 resulted from negativity primed in the context of collective temporal thought, and to examine whether the historical moment in which the original data were collected played a role. The hypothesis that the negativity bias in personal future thought resulted from engaging in personal temporal thought interleaved with collective temporal thought probes was not supported. It seems more plausible that the unusual negativity bias in personal future thought was a function of the historical moment in which the data were collected, in the context of the COVID-19 pandemic. Unfortunately, matched data from prior to the pandemic were not available, so we were not able to conduct the pre/post-test natural experiment that would enable us to make a more direct case for the pandemic’s causal role in reversing the typical positivity biases. However, interpreting our unusual data within the context of the existing literature, in which a baseline positivity bias is well-established, makes the historical moment interpretation plausible.

The lack of valenced bias in both autobiographical memory and personal future thought in Study 2 merits some contextualization. A negativity bias in personal future thought at the beginning of the pandemic followed by nonbiased personal future thought at the end of the pandemic suggests that there was a general increase in positivity across the two time points, even if there was not yet a return to the typically reported positivity bias. Such a return may require more temporal distance from the pandemic. Similarly, the positivity bias in autobiographical memory reported in Study 1 may have disappeared in Study 2 because negative personal events from the past 2 years may have been relatively salient. Unfortunately, events were not dated in the protocols, so this hypothesis cannot be tested directly with the current dataset.

## General discussion

The current studies were centrally concerned with the extent to which personal and collective temporal trajectories reflect one another, and the relation between implicit temporal trajectories and explicitly endorsed attitudes and beliefs. Although implicit trajectories of change, indexed as the change in valenced bias across two mentally represented temporal periods, did correlate between personal and collective referents in Study 1, this correlation was quite weak, and did not appear in Study 2. The lack of a correlation in Study 2 may have been due to a smaller sample in that study, failing to detect a weak correlation. In any case, implicit temporal trajectories for the personal self and nation seem to be largely, although perhaps not entirely, independent domains of temporal thought. This independence is particularly striking in that, for both British and American samples, data were collected at moments of high attention to national events and figures—for instance, Brexit in the UK and a highly polarizing president in the USA, as well as the COVID-19 pandemic in both countries. Future work ought to explore the question of whether personal and collective trajectories track one another more closely for other types of collectivities than the nation (e.g., families, workplaces, or other affiliative groups). It could be promising to examine this relation in the context of identity fusion, in which usually reciprocal personal and group identities fuse under conditions where the group-based threat of violence permeates everyday life (Atran, 2021). For instance, Reese and Whitehouse (2021) have put forward a model where negative/traumatic historical events can be narrated and ritualized, promoting identity fusion for young adults and adolescents.

As for the relation between asymmetries in cognitive availability and explicitly endorsed attitudes and beliefs, there do seem to be reliable relations between at least some explicitly endorsed attitudes and beliefs about the nation and valenced biases in collective temporal thought. Most relevant to our current purposes, implicit trajectories in collective temporal thought correlated most reliably with expressed hopefulness about the future of the nation. This finding is in line with MacLeod and Byrne’s (1996) report of a comparable correspondence in the domain of personal future thought, where negativity biases in personal future thought correlated with explicit feelings of hopelessness. The nature of this hope is likely nuanced; Bain et al. (2013) for instance suggested that people might be more motivated by a collective future characterized by better people (i.e., warmer and more moral) than by a future characterized by more favorable living conditions per se. Clearly, the connection between implicit trajectories and explicit beliefs, particularly hopefulness toward the future of the nation, is worthy of elaboration.

Other explicit attitudes correlated better with valenced biases within particular temporal domains, rather than changes across temporal domains. Across the two studies, the most reliable correlations were between collective future thought and beliefs about progress, decline, and shame in the nation’s history. Beliefs in progress and decline related primarily to collective future thought; their relation with collective memory was more tenuous, being statistically insignificant in Study 1 and significant but weak in Study 2. This suggests that beliefs in collective progress or decline are not inferred in relation to representations of the past, but as predictions about the future. However, the reliable negative correlation between shame in the nation’s past and collective future thought implies that shame in the nation’s history is associated with weaker positivity in collective future thought, and vice versa. Future work might explore the psychological nuances of these collective beliefs, and their preferential relation to different domains of collective temporal thought.

As a methodological caveat, our cognitive fluency measure on its own does not permit us to distinguish events that people believe are going to happen from events that come to mind easily for other reasons, but which participants do not actually strongly believe will occur. An important question for future work is how cognitive accessibility of collective events interacts with the perceived likelihood of those events, although basic research on the availability heuristic would suggest that people do often use cognitive availability as a basis for making judgments of probability (Tversky & Kahneman, 1973). There may be a cultural component to these judgments (Liu et al., 2012; Markus & Kitayama, 1991; Wang, 2016).

### Personal temporal thought

Both studies found an implicit downward trajectory in our participants’ mental representations of themselves as individuals extending across time, with negative events less accessible in autobiographical memory but predominating in imagination for the personal future. This pattern was pronounced in the early-pandemic sample, and remained, albeit in attenuated form, in the “post” pandemic sample. We should emphasize several points in interpreting this implicit trajectory of decline in personal temporal thought. First, it suggests that the valenced biases reported in prior literature do not apply in a blanket fashion across mentally represented temporal domains. That is, valenced biases need not be symmetrical between memory and future thought. Both valenced biases within a temporal domain and implicit trajectories across temporal domains may be systematically impacted by cognitive and social contexts. The personal past and future are represented in relation to one another, and a positively biased memory may reflect not a general mechanism for promoting positive self-regard, but a rosy past in relation to a future characterized by anxieties. It is necessary to interpret valenced biases in intertemporal contexts.

#### Age effects

In Study 1, age impacted valenced biases in personal temporal thought. Previous research reported that older people tend to have stronger positivity biases in autobiographical memory (Mather & Carstensen, 2005). In our Study 1 sample this correlation did not attain statistical significance, although the data pointed in the right direction numerically. However, personal future thought did decrease in positivity with age. This may be explained by the fact that older adults occupy a location in the life script where negative events tend to predominate, rather than a generally negative disposition per se (Durbin et al., 2019). That is, when imagining the future—the temporal frame for which we did not specify—participants of different ages may have been imagining different periods. Given that negative events in life scripts are more common toward the end than the beginning or middle, older participants using a life script to help scaffold future imagination would have retrieved more negative events. The lack of any age effects in Study 2, however, should encourage caution in interpreting these findings.

One additional note on our American and British samples. These samples were designed to provide a replication and extension of previous research on intertemporal trajectories, which have been largely restricted to participants from Western populations. Cross-cultural psychology (e.g., Markus & Kitayama, 1991) suggests that positivity biases in general are less prevalent in non-Western societies. Little research has explicitly examined cultural variability in intertemporal representations and implicit trajectories concerning the self across cultures (however, see Deng et al., 2022, for some suggestive early work in Chinese and American samples). Until such programmatic work has progressed further, we suggest remaining cognizant of the possibility that the effects reported are culturally bound, particularly since the content and general structure of at least one component of personal temporal thought, autobiographical memory, varies considerably between cultures (Wang, 2004, 2016).

### Collective temporal thought

The current studies demonstrated an implicit trajectory of national decline in a British sample, adding the UK to the population of Western countries in which this cognitive phenomenon has been observed. This implicit trajectory is related to, but separate from, the more general negativity bias in collective temporal thought. As with personal temporal thought, we advise caution in universalizing these findings, given that little research in collective temporal thought has been done in non-Western populations. Some early findings in Chinese and Turkish samples suggest collective temporal thought may indeed be culturally variable, although negativity biases do seem common (Deng et al., 2022; Hacibektasoglu et al., 2022; Mert et al., 2022). Living historical memories in Western countries tends to be more negative than in developing countries (Choi et al., 2021). These data suggest that our findings may reflect a perceived trajectory of decline in Western countries relative to other parts of the world (Liu et al., 2014), as opposed to being a stable, universal constant. Western historiography from the 18^th^ century onwards has emphasized progress predicated on an enlightenment narrative (Iggers et al., 2008; Liu & Robinson, 2016), but this narrative of progress appears to have collapsed into a more pessimistic view in recent years (Nora, 1996; Páez et al., 2016; Schwartz, 2008).

## Declarations

This research was approved by the Massey University Human Ethics Committee and UCSC Human Research Protections Program. The data and materials reported here are publicly available on the Open Science Framework, under the project Implicit National Trajectories (https://osf.io/n8329/). This research was not preregistered.
